# Altered White Matter Microstructure in Herpes Zoster and Postherpetic Neuralgia Determined by Automated Fiber Quantification

**DOI:** 10.3390/brainsci12121668

**Published:** 2022-12-04

**Authors:** Ying Wu, Lili Gu, Shunda Hong, Jiahao Li, Jiaojiao Yang, Jiaxin Xiong, Huiting Lv, Jian Jiang

**Affiliations:** 1 Department of Radiology, The First Affiliated Hospital, Nanchang University, Nanchang 330006, China; 2 Neuroimaging Laboratory, Jiangxi Province Medical Imaging Research Institute, Nanchang 330006, China; 3 Department of Pain Clinic, The First Affiliated Hospital, Nanchang University, Nanchang 330006, China

**Keywords:** neuropathic pain, white matter, automated fiber quantification, diffusion tensor imaging

## Abstract

This study aimed to explore changes in the white matter microstructure in herpes zoster (HZ) and postherpetic neuralgia (PHN) patients and to estimate the correlation of these changes with clinical data. Diffusion tensor imaging (DTI) data were collected from 33 HZ patients, 32 PHN patients, and 35 well-matched healthy controls (HCs). Subsequently, these data were analyzed by automated fiber quantification (AFQ) to accurately locate alterations in the white matter microstructure. Compared with HCs, HZ and PHN patients both showed a wide range of changes in the diffusion properties of fiber tracts. HZ patients exhibited changes primarily in the left superior longitudinal fasciculus (SLF), whereas PHN patients predominantly exhibited changes in the left inferior fronto-occipital fasciculus. The bilateral SLF and the left corticospinal tract were altered in the PHN patients compared with HZ patients. In addition, PHN patients showed a trend toward more expansive white matter alterations compared with those observed in HZ patients; additionally, in PHN patients, changes in the left cingulum cingulate were significantly correlated with changes in emotion and the duration of disease. These findings may help to elucidate the transformation from HZ to PHN and provide new ideas regarding the reasons for intractable neuropathic pain in PHN.

## 1. Introduction

Herpes zoster (HZ), caused by reactivation of the latent varicella-zoster virus (VZV) in the spinal cord or cranial sensory ganglia, is typically characterized by a unilateral acute painful rash of the affected skin, which is self-limiting and generally disappears within a few weeks [1]. Postherpetic neuralgia (PHN) is the most common clinical complication of HZ and presents with severe neuropathic pain, usually persisting for more than 1 month after the rash heals, which is in line with Chinese expert consensus [2]. A study showed that the prevalence rates of HZ and PHN are 7.7% and 2.3%, respectively [2]. Among HZ patients, 29.8% developed PHN, and the prevalence of both HZ and PHN tends to increase with age. These patients suffer from chronic pain, which seriously affects their quality of life and imposes a burden on society [3].

The pathogenesis of PHN is not yet fully understood. Currently, PHN is thought to be caused by abnormally high excitability of pain-related neurons in the spinal cord and above the spinal cord or by enhanced synaptic transmission, thereby amplifying the central sensitization mechanism of pain signal transmission, leading to neuropathic pain for an extended period in patients with HZ and PHN [1]. Moreover, dorsal root ganglia atrophy seen in PHN may arise due to direct infection of the spinal cord or transsynaptic degeneration [4]. Studies have shown that this kind of neuropathic pain remodels brain structure and function [5], and more studies have explored altered brain function in patients with HZ and PHN [6,7,8]; however, research on the brain structure of patients with PHN is relatively limited. Of note, Chen et al. found decreased fractional anisotropy (FA) and axial diffusivity (AD) of the white matter in the insula, anterior central gyrus, cerebellum, occipital lobe, and other regions in PHN patients [9]. In a diffusion kurtosis imaging (DKI) study, Zhang et al. found that DKI parameters of the left middle frontal gyrus and occipital lobe, bilateral insula and superior temporal gyrus, and right anterior cerebellar lobe were significantly reduced in PHN patients compared with those in healthy controls [10]. In addition, a longitudinal study of PHN patients before and after treatment showed that the AD of the right thalamus in PHN patients decreased significantly after treatment, and the FA of the right superior temporal gyrus increased greatly [11]. These results suggest that the white matter microstructure of PHN patients is influenced by long-term pain. However, whether similar changes occur in HZ patients and how these changes manifest require further study.

Diffusion tensor imaging (DTI), a noninvasive method of central nervous system imaging, can fully characterize diffusion anisotropy by estimating the diffusion characteristics of water molecules in brain tissues; this characterization indicates the connectivity of white matter tracts and provides more detailed information about the tissue microstructure. Various methods have been developed to analyze DTI data, including voxel-based analysis (VBA) and tract-based spatial statistics (TBSS), which both utilize voxel-based analysis as the registration algorithm cannot accurately pinpoint the actual position of the fiber tract in individual brains [12,13]. However, tractography, which reconstructs white matter tracts through algorithms that estimate the orientation distribution function, is widely considered the most accurate method for identifying white matter fiber fascicles in the living human brain [14,15]. Notably, automatic fiber quantification (AFQ) is a method based on tractography that can quickly and reliably identify 20 white matter tracts in the brain; this method can be used to further analyze the diffusion characteristics of anatomically equivalent positions along the fiber track. Since neuropathic pain can manifest at specific positions along the different fiber tracts [16], AFQ provides an opportunity to investigate the local specificity of white matter fiber changes in HZ and PHN patients. Therefore, the purpose of this study was to explore alterations in the white matter microstructure in HZ and PHN patients by AFQ and to investigate whether these diffusion indices are significantly correlated with clinical data.

## 2. Materials and Methods

### 2.1. Subjects

The subjects were collected from March 2020 to February 2022 from the Pain Department of the First Affiliated Hospital of Nanchang University. We originally included HZ (52), PHN (48), and HC (39) patients. After MRI scanning, we screened out 39 subjects with poor image quality (e.g., obvious head movement, motion artifact, and preprocessing failure). Therefore, 33 HZ (age: 62.97 ± 9.99 years), 32 PHN (age: 64.47 ± 10.14 years), and 35 HC (age: 62.49 ± 4.39 years) patients were ultimately included in our study. We used Gpower 3.1.1 software [17] to justify our sample size. The inclusion criteria for patients with HZ and PHN were as follows: (1) right-handed; (2) diagnosed with HZ (skin lesions that persist for longer than 2 weeks) or PHN (pain persisting for more than one month after the HZ rash has healed [2]) according to the diagnostic criteria defined by the International Association for the Study of Pain [2] by two clinically experienced physicians in the pain department; and (3) pain scores on a visual analog scale (VAS) ≥ 5 points. The exclusion criteria were as follows: (1) other chronic pain or neurological diseases; (2) special types of HZ, such as in the eyes or ears; and (3) MRI contraindications. The exclusion criteria for HCs were as follows: (1) left-handed; (2) self-reported negative emotions such as anxiety or depression and neurological diseases; (3) chronic pain and contraindications to MRI. The skin sites where herpes zoster occurred in most HZ and PHN patients were located on the anterior and posterior thoracic areas.

Before the MRI scan, HZ and PHN patients indicated their pain intensity on a VAS scored from 0 (no pain) to 10 (the most intolerable pain). The Hamilton Depression Scale (HAMD) [18] and Hamilton Anxiety Scale (HAMA) [19] were used to assess the degrees of depression and anxiety, respectively. Psychological or emotional disorders were assessed using scores on the Symptom Checklist-90-Revised (SCL-90-R) [20]. However, the HCs were not assessed with HAMA and HAMD since we have excluded subjects with self-reported negative emotional distress.

### 2.2. Image Acquisition

MRI data were acquired using a 3.0 T Siemens TIM Trio scanner (Erlangen, Bavaria, Germany) in the Radiology Department of the First Affiliated Hospital of Nanchang University. Three different sequences were used as follows. (1) For DTI diffusion-weighted images, a single echo planar imaging sequence with 30-way diffusion coding (b = 1000 s/mm^2^ for each direction) and no diffusion coding (b = 0 s/mm^2^) was used. The parameters of image acquisition were as follows: repetition time (TR) = 6600 ms, echo time (TE) = 102 ms, field of view (FOV) = 256 × 256 mm^2^, slice thickness = 2 mm, slice spacing = 0.2 mm, and matrix = 128 × 128. (2) For three-dimensional high-resolution T1-weighted images of 176 sagittal slices for each subject, the following parameters were used: TR = 1900 ms, TE = 2.26 ms, flip angle = 9°, matrix = 240 × 256, FOV = 215 × 230 mm^2^, and slice thickness = 1 mm. (3) Conventional T2-weighted imaging was used to rule out visible brain structural abnormalities. All subjects were instructed to lay quietly during MRI acquisition and provided earplugs to reduce the noise from the MRI machine.

### 2.3. AFQ Analysis

The raw MRI data were preprocessed by FSL 5.0.9 (FMRIB Software Library, University of Oxford, UK) [21]. The FMRIB Linear Image Registration Tool was applied for affine coregistration of every diffusion-weighted image to the image of b0 to correct for image distortion caused by eddy current changes and head motion [22]. Next, the transformation matrix was extracted, and the diffusion gradient direction was rotated. The Brain Extraction Tool (BET) command was run to obtain brain masks of T1 and b0 images with a fractional intensity threshold of 0.2. The diffusion tensor model was constructed with the amended diffusion gradient direction matrix using FMRIB’s diffusion tool (FDT) to reconstruct the diffusion tensor, and images of FA, mean diffusivity (MD), AD, and radial diffusivity (RD) were produced simultaneously.

After obtaining the preprocessed images, the white matter fiber tracts of the brain were analyzed using AFQ software [16], which is an open-source software based on MATLAB that automatically identifies 20 major fiber tracts referring to the identification method presented by Hua et al. [23], who proposed that the diffusion tensor should be mapped to the finely segmented tracts. The detailed identification process of AFQ is as follows. First, deterministic fiber tracking imaging of whole-brain fiber is performed by applying a streamlined tracking algorithm using a fourth-order Runge–Kutta path integration to track the fibers in the whole brain mask [24]. Second, the fiber tracts were segmented and refined by a predefined ROI method proposed by Wakana et al. [25] to separate every fiber tract from the whole brain fiber tracts; the obtained fiber bundles were combined with the JHU white matter tractography to refine the fiber bundles. Third, the outliers, which deviated (>5 standard deviations) from the core of the tract or were longer (>4 standard deviations) than the mean fiber length based on the Gaussian distribution, were removed. Fourth, each tract was cut into a central part of the two waypoint ROIs to keep individual level consistency. Fifth, the diffusion tensor was calculated for fiber-tract quantification. Therefore, 100 nodes of 20 main tracts were acquired. The 20 tracts are as follows: the bilateral anterior thalamus radiation (ATR), corticospinal tract (CST), cingulum cingulate (CGC), cingulum hippocampus (CGH), inferior fronto-occipital fasciculus (IFOF), inferior longitudinal fasciculus (ILF), superior longitudinal fasciculus (SLF), uncinated fasciculus (UF), arcuate fasciculus (AF), and callosum forceps major (posterior forceps of the corpus callosum, CCF_P) and callosum forceps minor (anterior forceps of the corpus callosum, CCF_A).

### 2.4. Statistical Analysis

SPSS 26.0 (SPSS Inc., Chicago, IL, USA) software was used for statistical analyses. Continuous variables were tested for normality using the Shapiro–Wilk test. The mean ± standard deviation is reported for continuous variables with a normal distribution. Continuous variables, such as age, VAS score, SCL-90-R score, HAMA score, and HAMD score, were compared by analysis of variance (ANOVA) in the patients and control groups, and sex ratios were compared using Pearson’s chi-square test. All statistical assessments were two-tailed with significance thresholds of *p* < 0.05.

Yeatman et al. [26] proposed that local changes in fibers could be assessed entirely by a point-by-point comparison of extracted tract profiles. A group-level point-by-point analysis of the diffusivity index at 100 points of each fiber tract was performed. After age and sex were included as covariates, one-way ANOVA was conducted on the mean diffusion characteristics of 20 fiber tracts and each point-by-point diffusion measure (FA, MD, RD, and AD) to determine differences among groups; multiple comparisons were corrected according to the false discovery rate (FDR, *q* < 0.05). The points with 3 or more consecutive significant nodes were retained, and the differences among the three groups were then analyzed using post hoc tests. Pearson correlation analysis was used to explore the relationship between clinical data and fiber-tract diffusion characteristics.

## 3. Results

### 3.1. Group Differences in Demographic and Clinical Characteristics

No significant differences in age (*p* = 0.617), sex (*p* = 0.867) were found among the three groups. VAS scores (*p* = 0.674) and HAMD scores (*p* = 0.084) were not significantly different between the HZ and PHN groups, while there were significant differences in the duration of disease (*p* = 0.021), SCL-90-R scores (*p* = 0.006) and HAMA scores (*p* = 0.044) (Table 1).

### 3.2. Group Differences in Mean Diffusion Properties

The numbers of subjects in whom the 20 fiber tracts were successfully reconstructed in three dimensions by AFQ among the three groups are displayed in column “n1:n2:n3”, which represents the numbers of subjects from the HZ, PHN, and HC groups, respectively (Table 2, Table 3, Table 4 and Table 5). The mean diffusion measurements (FA, MD, AD, and RD per tract) were compared among groups at the whole-brain level.

Regarding FA, the FA of the left CGC in the HZ and PHN groups was significantly lower than that in the HC group, the FA of the right CGC in the PHN group was significantly lower than that in the HC group, and the FA of the left SLF in the HZ group was higher than that in the HC group.

Regarding MD, the MD of the left CST, left CGC, and left SLF in the HZ and PHN groups were significantly higher than those in the HC group. The MD of the right CGC and left SLF in the PHN group were significantly higher than those in the HC group.

Regarding RD, the RD of the bilateral CGC in the HZ and PHN groups was significantly higher than that in the HC group, and the RD of the right ILF in the PHN group was significantly higher than that in the HC group.

Regarding AD, the AD of the left SLF and left AF in the HZ and PHN groups were significantly higher than that in the HC group, and the AD of the left CST in the PHN group was significantly higher than that in the HC group.

### 3.3. Group Differences in the Regional Distribution of Diffusion Properties

**Figure 1:**
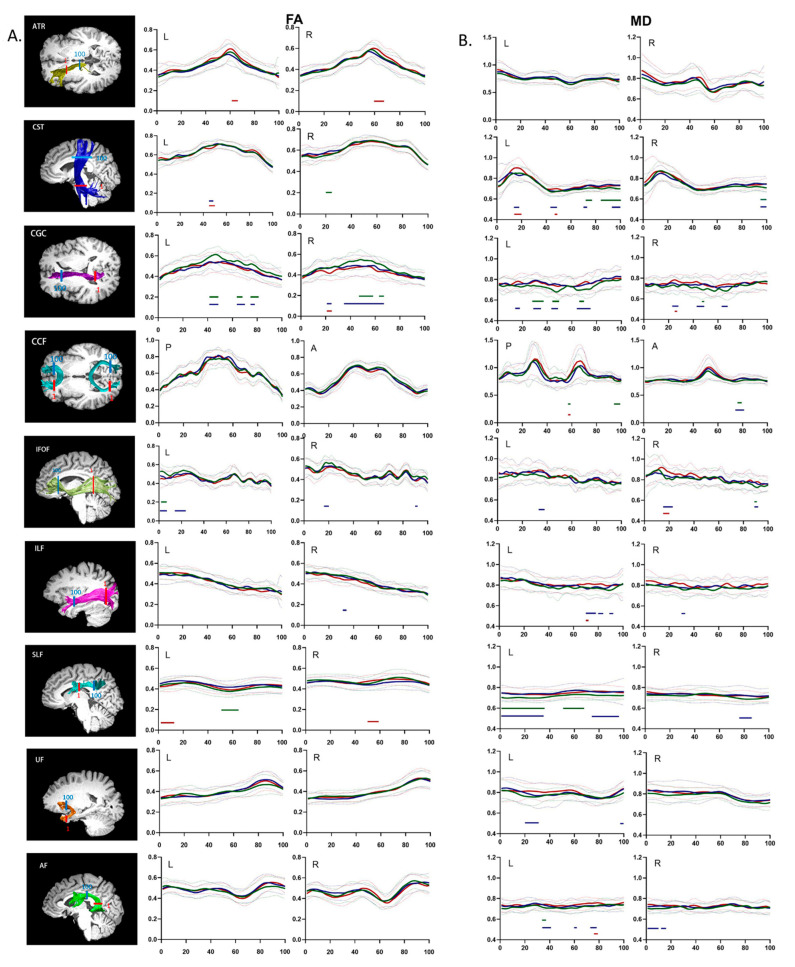
Pointwise comparison of (**A**) fractional anisotropy (FA) and (**B**) mean diffusivity (MD) profiles among the HZ, PHN, and HC groups. Notes: The renderings of significantly altered tracts are presented on the T1-weighted images on the left. Red: starting ROIs; blue: ending ROIs. The FA and MD profiles of 20 identified fiber tracts from HZ patients, PHN patients, and HCs (blue, red, and green, respectively) are presented as the mean (SD) (solid line and dotted line, respectively). The color bars under the FA or MD profiles indicate the regions of significant differences between the HZ and HC groups (green), the PHN and HC groups (blue), and the HZ and PHN groups (red). The *Y*-axis represents the FA or MD values. The *X*-axis represents the location between the starting and ending waypoint ROIs. Abbreviations: P, posterior; A, anterior; L, left; R, right.

**Figure 2:**
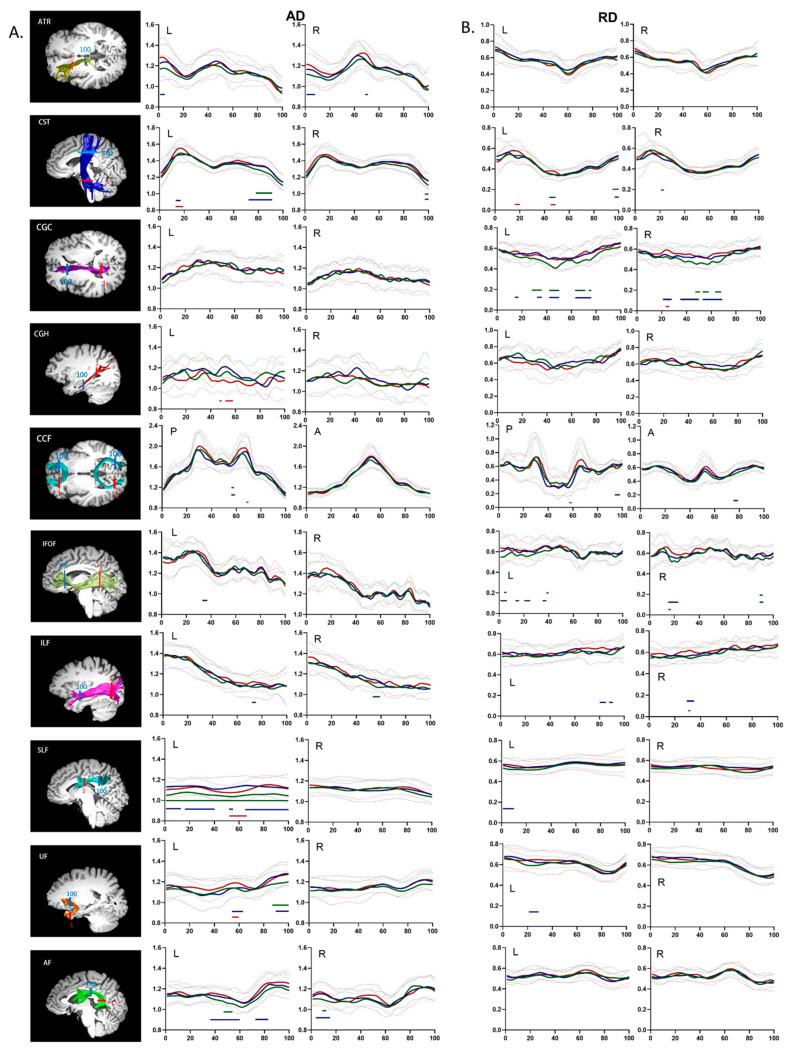
Pointwise comparison of (**A**) axial diffusivity (AD) and (**B**) radial diffusivity (RD) profiles among the HZ, PHN, and HC groups. Notes: The renderings of significantly altered tracts are presented on the T1-weighted images on the left. Red: starting ROIs; blue: ending ROIs. The FA and MD profiles of 20 identified fiber tracts from HZ patients, PHN patients, and HCs (blue, red, and green, respectively) are presented as the mean (SD) (solid line and dotted line, respectively). The color bars under the FA or MD profiles indicate the regions of significant differences between the HZ and HC groups (green), the PHN and HC groups (blue), and the HZ and PHN groups (red). The *Y*-axis represents the AD or RD values. The *X*-axis represents the location between the starting and ending waypoint ROIs. Abbreviations: P, posterior; A, anterior; L, left; R, right.

Unlike conventional fiber-tract imaging, AFQ analysis can quantify diffusion measurements along the white matter fiber tract to provide detailed diffusion characteristics. Dotted comparisons of the diffusion characteristics among the HZ, PHN, and HC groups are shown in Figure 1 (FA and MD) and Figure 2 (AD and RD). Multiple comparisons among all 100 points per fiber tract were corrected by FDR-adjusted *p* values < 0.05 to reduce Type I errors.

The pointwise comparison of FA revealed that HZ patients showed extensive FA decreases in the inferior portion of the right CST, the anterior and medial portions of bilateral CGC, the posterior portion of the left IFOF, and the posterior portion of the left SLF compared to HCs. Compared with HCs, PHN patients had decreased FA in the middle portion of the left CST, the anterior and middle portions of the left CGC, the anterior, middle, and posterior portions of the right CGC, the posterior portion of the left IFOF, the anterior and posterior portions of the right IFOF, and the middle portion of the right ILF. Compared with the HZ group, PHN patients had decreased FA in the posterior portion of the bilateral ATR, the medial portion of the left CST, the posterior portion of the right CGC, the anterior portion of the left SLF, and the posterior portion of the right SLF.

The pointwise comparison of MD showed that compared to HCs, HZ patients had substantial increases in MD in the superior portion of the bilateral CST; the anterior, middle, and posterior portions of the left CGC; the middle portion of the right CGC; the left portion of the anterior and posterior CCF; the anterior portion of the right IFOF; the anterior, middle, and posterior portions of the left SLF; and the middle of the portion of the left AF. Additionally, compared to HCs, PHN patients had increased MD in the following regions: the superior, middle, and inferior portions of the left CST; the superior portion of the right CST; the anterior, middle, and posterior portions of the bilateral CGC; the left portion of the anterior CCF; the middle portion of the left IFOF; the anterior and posterior portions of the right IFOF; the anterior portion of the left ILF; the middle portion of the right ILF; the anterior, middle, and posterior portions of left SLF; the posterior portion of the right SLF; the frontal and temporal lobe portions of the left UF; the middle and frontal lobe portions of the left AF; and the temporal portion of the right AF. Compared to the HZ group, PHN patients showed increased MD in the middle and inferior portions of the left CST, the posterior portion of the right CGC, the left portion of the posterior CCF, the posterior portion of the right IFOF, the anterior portion of the left ILF, and the frontal lobe portion of the left AF.

The pointwise comparison of AD demonstrated that compared to HCs, HZ patients showed substantial increases in AD in the superior portion of the bilateral CST, the middle portion of the right ILF, the entire left SLF, the frontal lobe portion of the left UF, the middle portion of the left AF, and the temporal lobe portion of the right AF. Compared with HCs, PHN patients showed substantially increased AD in the anterior portion of the left ATR, the anterior and middle portions of the right ATR, the superior and inferior portions of the left CST, the superior portion of the right CST, the middle portion of the left IFOF, the anterior portion of the left ILF, the anterior, middle, and posterior portions of the left SLF, the middle and frontal lobe portions of the left UF, the middle and frontal lobe portions of the left AF, and the temporal lobe portion of the right AF. Compared to the HZ group, PHN patients showed substantially increased AD in the inferior portion of the left CST, the middle portion of the left CGH, the middle portion of the left SLF, and the middle portion of the left UF.

Pointwise comparison of RD revealed that compared to HCs, HZ patients showed substantially increased RD in the superior portion of the left CST; the inferior portion of the right CST; the anterior, middle, and posterior portions of the left CGC; anterior and middle portions of right CGC; the left portion of the posterior CCF; the middle and posterior portions of the left IFOF; and the anterior portion of the right IFOF. Compared with HCs, PHN patients showed substantially increased RD in the anterior and middle portions of the left CST; the anterior, middle, and posterior portions of the bilateral CGC; the left portion of the anterior CCF; the middle and posterior portions of the left IFOF; the anterior and posterior portions of the right IFOF; the anterior portion of the left ILF; the middle and posterior portions of the right ILF; the anterior portion of the left SLF; and the temporal lobe portion of the left UF. Compared to the HZ group, PHN patients showed substantially increased RD in the middle and inferior portions of the left CST, the posterior portion of the right CGC, the left portion of the posterior CCF, the posterior portion of the right IFOF, and the posterior portion of the right ILF.

### 3.4. Correlation Analysis

**Figure 3:**
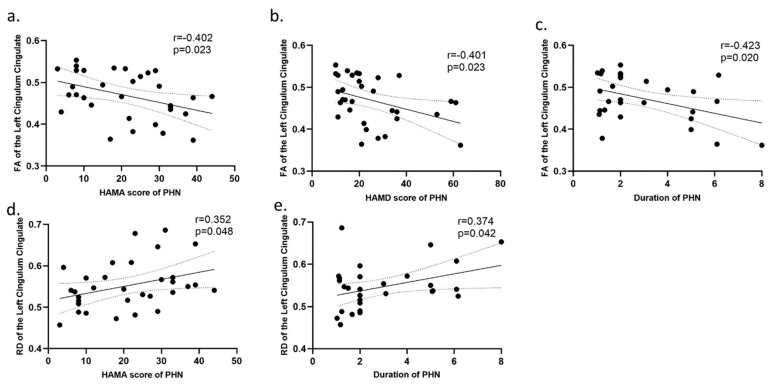
Correlation analysis between the diffusion properties and the clinical characteristics (**a**–**e**). Abbreviations: HZ, herpes zoster; PHN, postherpetic neuralgia; fractional anisotropy (FA); radial diffusivity (RD); HAMA, Hamilton Anxiety Scale; HAMD, Hamilton Depression Scale.

In the analysis of the correlations between clinical characteristics and average diffusion measurements (FA, MD, AD, and RD) of the significantly altered fiber tracts in the PHN and HZ groups, it was found that the FA of the left cingulum cingulate gyrus in the PHN group was negatively correlated with HAMA scores, HAMD scores, and the duration of disease; the RD of the left cingulum cingulate was positively correlated with HAMA scores and the duration of disease. There were no significant correlations among the variables in the HZ group (Figure 3).

## 4. Discussion

AFQ can more accurately locate tract segments that differ among groups by tracking 20 fiber tracts in the whole brain and segmenting them at equal intervals of 100 points, thereby enabling assessment of the integrity of white matter fiber tracts in participants in the HC, HZ, and PHN groups. This method revealed a wide range of changes in the diffusion properties of fiber tracts among the three groups. Specifically, the differences between the HZ and PHN groups mainly manifested as decreased FA of the bilateral SLF, increased MD of the left CST, increased AD of the left CGH and left SLF and increased RD of the left CST. Compared with HCs, the FA of the bilateral CGC and left SLF in the HZ group were decreased to different extents, while increases in the MD, RD, and AD were mainly observed in the left CST, left CGC, and left SLF. The abovementioned properties were also increased in some segments of the right CGC, left IFOF, and left UF. In PHN patients, more extensive changes in the diffusion properties of the fiber tracts were observed. A decrease in the FA was observed in the bilateral CGC and left IFOF. Increases in the MD, RD, and AD were observed in many fiber tracts, such as the left CGC, left SLF, left UF, and left AF. As HZ progressed to PHN, the abnormalities in some white matter fiber tracts expanded. For example, the left CST showed increased MD in the superior portion in HZ patients. However, in PHN patients, increased MD was also observed in the middle and inferior portions of the left CST. The areas with altered tract diffusion properties (FA, MD, AD, and RD) of the right CGC expanded (Table 6 and Table 7). This expansion suggests that the persistent pain experienced by PHN patients might be related to the aggravation of injury to these specific fiber tracts.

White matter, which comprises of axons coated with myelin sheaths, allows conduction of signals between neurons and coordinates signals among brain regions during normal operations [27]. The gray matter (comprised of neuron bodies) is thus the basis of white matter fibers. Recent studies have reported changes in cortical thickness and gray matter volume in patients with HZ and PHN compared with normal control groups [8,28]. The peripheral and central neural networks related to nociception processing pathways show extensive plasticity in chronic neuropathic pain. This plasticity manifests as changes in individual molecules, synapses, cellular function, at the structural level [29]. Reconstruction of the white matter fiber tract by DTI, which examines the movement of water molecules through and along the fiber tract, can characterize the fiber-tract diffusion properties, thus describing abnormalities in the white matter microstructure, such as the morphology and density of axons, the degree of myelin coverage, and changes in water content [30,31]. For example, according to Beaulieu et al., the FA plays an important role in assessing axonal membrane structures, and a decrease in the FA may mean that the fiber tracts become looser [32]. Increases in the MD may indicate the aggravation of demyelination or fiber tract edema. The AD measures the diffusion rate parallel to the fiber tract, which is related to axonal integrity [33]. Furthermore, the RD can be used to measure the diffusion rate perpendicular to the fiber tract. Relatedly, Brusini et al. found that the RD can be used to indicate changes in myelin sheath content [34]. Our findings reveal widespread degradation of white matter tracts in patients with HZ and PHN, and the inconsistent results observed in the measured diffusion indicators may indicate differences in the severity of disruption between these regions. Among the significantly changed fiber tracts (the CST_L, CGC, SLF_L, IFOF_L, and AF_L), there were widespread reductions in the FA and widespread elevations in the MD, AD, and RD. Moreover, against the background of FA and MD alterations, the changes in the AD of some fiber tracts were more obvious than those in the RD. For example, the MD of the middle portion of the left SLF exhibited significant alterations in the HZ and PHN subjects, and HZ patients appeared to have changes in the overall fiber tract (AD of points 1–100) and most of the segments changed (AD of points 1–13, 16–40, 52–55, and 65–100) in PHN patients, while the RD of HZ patients (no points) and PHN patients (points 1–10) did not obviously change. A similar phenomenon was observed in the left UF of the HZ group and the left UF and bilateral AF of the PHN group, suggesting that the degradation of these fiber tracts may be mainly caused by axonal degeneration. However, the bilateral CGC and left IFOF of the HZ group and the bilateral CGC and bilateral IFOF of the PHN group exhibited more pronounced RD alterations, suggesting greater degradation of the myelin sheath in these fiber tracts. Additionally, the regions of axon and myelin-sheath degradation were further increased in patients with PHN compared with those with HZ, again supporting the above view that the damage to the white matter microstructure in PHN patients is aggravated compared with that in patients with HZ. Previously, a DTI study of PHN revealed changes in the FA and AD [9] and it was suggested that the changes in DTI parameters in PHN patients were due to the degradation of axons rather than axon demyelination or other reasons. Despite the consistent conclusions of our study, we believe that the main reason for fiber damage may vary among tracts and that the degradation of the myelin sheath also has a significant effect. Some studies of animal models of PHN have shown that when the HZ virus reactivates in the ganglia, it not only leads to the degradation of the axons responsible for pain transmission but also causes damage to satellite glial cells and Schwann cells, resulting in myelin sheath loss and other phenomena [35,36]. Additionally, there is a unique T-junction in dorsal root ganglia, which can enhance the signal from the periphery toward the central nervous system after injury to the peripheral axon, causing ectopic discharges and axonal damage in the central nervous system [37]. In addition, long-term pain can affect the function and chemical profile of neurons by activating, regulating, and modifying primary sensory neurons and dorsal horn neurons, thereby affecting neuroplasticity and widely affecting brain structure [38].

Studies have shown that the cingulate fasciculus connects the frontal lobe, parietal lobe, and temporal lobes [14]; this tract, along with the prefrontal lobe cortex, amygdala, anterior cingulate cortex, hippocampus, and insula, forms the limbic system. The cingulate fasciculus, the core link among these regions, plays an important role in executive function, emotions, pain perception, and so on [39]; in addition, the cingulum hippocampus plays an important role in episodic memory [40]. Our study revealed extensive DTI index abnormalities were found in the bilateral CGC, the CGH was more difficult to track and showed few changes, but comparison of the HZ and PHN groups revealed increased AD in some segments of the left CGH. This finding was consistent with the results of previous TBSS and DKI studies [9,10], suggesting that the abnormal processing of emotion and pain in patients with HZ and PHN might be related to changes in the microstructure of the bilateral CGC. In addition, the central anterior gyrus of the motor cortex and the somatomotor cortex, which are dominated by the CST, play an important role in the perception and regulation of pain [41]. A recent study found that in PHN patients, the thickness of the motor cortex decreases, and the primary somatosensory cortex expands accordingly [28]. Another study combining DTI and resting-state MRI revealed microstructural changes in brain regions, such as the anterior central gyrus, in patients with PHN [42]. In addition, microstructural changes have been reported in different segments of liaison fibers, such as the SLF, ILF, UF, and IFOF. The temporal-parietal-occipital region, a complex brain region formed by these fiber tracts, participates in or coordinates many important brain functional activities, such as language, vision, working memory, and recognition [43]. Many previous studies revealed changes in gray matter volume and cortical thickness in the temporal, parietal, and occipital lobes of the brain in PHN patients [8,28,44]. This finding indicates that HZ and PHN patients demonstrate extensive changes in brain structure, which may be related to the inflammatory response and prolonged activation of glial cells, which contribute to the modification of the central nervous system [38,45].

In our study, the FA of the left CGC in PHN subjects was negatively correlated with HAMA scores, HAMD scores, and the duration of disease. Moreover, the RD was positively correlated with HAMA scores and the duration of disease, suggesting that the integrity of the white matter might be related to the generation of negative emotions and worsened with the continuation of pain. Many studies have shown that the cingulate fasciculus is key for emotional processing and critically contributes to patterned activities associated with pain perception [46,47,48]. This phenomenon was more obvious in the PHN group because these patients endured long-term pain. Studies have shown that in chronic pain, such as that experienced by trigeminal neuralgia patients, negative emotions and anxiety disorders are associated with cingulate fasciculus activity because the cingulate fasciculus is one of the components of the emotional circuit, and it plays an important role in emotion regulation [49]. Therefore, it is reasonable to speculate that negative emotions, anxiety, and depression in patients with HZ and PHN may further aggravate the damage to the white matter fiber tract.

However, this study has some limitations. First, as HCs were not assessed with HAMA and HAMD, there is insufficient evidence on whether HCs are completely free of anxiety and depression during MRI scanning, and the correlation between white matter integrity changes and anxiety and depression is not clear in HCs. We will collect the scale of each subject to improve our study in future studies. Second, since some fibers, such as the bilateral cingulate hippocampus, are adjacent to gray matter, the threshold setting for fiber tracking and the FA in this portion is low and may lead to partial fiber tracking failure. Third, although we performed a multipoint analysis with age as a covariate and correction for multiple comparisons, the effect of age on white matter hyperintensities should be taken into account. Fourth, the design of this study was a cross-sectional design. Therefore, we could not determine the causal dynamic mechanisms by which white matter alterations in HZ patients developed into those in PHN patients, and more longitudinal studies are required to further explore the underlying mechanisms.

## 5. Conclusions

This study revealed that patients with HZ and PHN exhibit extensive changes in the white matter microstructure compared with healthy people, and these alterations were more prominent in the PHN group than in the HZ group. Our findings help to elucidate the pathogenesis of the transition from HZ to PHN and provide theoretical support for the early identification of and clinical intervention in PHN patients.

## Figures and Tables

**Table 1:** Clinical and demographic characteristics of the subjects.

	HZ (n = 33)	PHN (n = 32)	HC (n = 35)	F-Value	*p*-Value
Age (years)	62.97 ± 9.99	64.47 ± 10.14	62.49 ± 4.39	0.49	0.617
Sex (M/F)	Age (years)	18/14	18/17	—	0.867
VAS score	Sex (M/F)	6.38 ± 1.04	—	0.18	0.674
Duration of disease (months)	VAS score	6.87 ± 15.80	—	5.58	0.021
SCL-90-R score	Duration of disease (months)	147.13 ± 39.48	—	8.00	0.006
HAMA score	SCL-90-R score	21.00 ± 11.82	—	4.22	0.044
HAMD score	HAMA score	25.25 ± 15.30	—	3.08	0.084
	HAMD score				

Notes: Data are presented as the means ± standard deviations. Abbreviations: HZ, herpes zoster; PHN, postherpetic neuralgia; HC, healthy control; M/F, male/female; HAMA, Hamilton Anxiety Scale; HAMD, Hamilton Depression Scale; VAS, visual analog scale. F-values and *p*-values represent ANOVA test results.

**Table 2:** Comparison of mean FA among the three groups.

	n1:n2:n3	HZ	PHN	HC	ANOVA		Post Hoc Groupwise (FDR)		
		(N = 33)	(N = 32)	(N = 35)	F-Value	p-Value	HZ vs. HC	PHN vs. HC	HZ vs. PHN
ATR_L	33:31:35	0.431 ± 0.042	0.450 ± 0.037	0.440 ± 0.044	1.795	0.172	0.359	0.313	0.061
ATR_R	32:32:35	0.443 ± 0.036	0.454 ± 0.036	0.443 ± 0.040	0.892	0.413	0.947	0.232	0.269
CST_L	33:32:35	0.621 ± 0.031	0.622 ± 0.031	0.622 ± 0.032	0.008	0.992	0.903	0.968	0.937
CST_R	33:32:35	0.615 ± 0.031	0.606 ± 0.028	0.608 ± 0.033	0.831	0.439	0.296	0.882	0.243
CGC_L	32:32:32	0.468 ± 0.048	0.469 ± 0.055	0.501 ± 0.059	3.819	**0.025**	**0.017**	**0.020**	0.954
CGC_R	31:32:31	0.443 ± 0.051	0.426 ± 0.050	0.465 ± 0.040	5.302	**0.007**	0.066	**0.002**	0.174
CGH_L	13:10:11	0.366 ± 0.042	0.365 ± 0.049	0.357 ± 0.043	0.150	0.861	0.615	0.667	0.966
CGH_R	19:23:18	0.368 ± 0.042	0.370 ± 0.035	0.375 ± 0.045	0.171	0.843	0.566	0.702	0.825
CCF_P	33:32:35	0.609 ± 0.057	0.599 ± 0.047	0.599 ± 0.062	0.333	0.718	0.482	0.984	0.480
CCF_A	33:32:35	0.521 ± 0.044	0.514 ± 0.048	0.532 ± 0.039	1.505	0.227	0.295	0.090	0.514
IFOF_L	33:32:35	0.453 ± 0.043	0.448 ± 0.031	0.465 ± 0.032	1.920	0.152	0.173	0.063	0.617
IFOF_R	33:31:33	0.456 ± 0.037	0.453 ± 0.039	0.465 ± 0.033	0.988	0.376	0.302	0.185	0.754
ILF_L	33:31:34	0.411 ± 0.034	0.411 ± 0.036	0.417 ± 0.033	0.404	0.669	0.454	0.430	0.954
ILF_R	32:32:35	0.417 ± 0.031	0.405 ± 0.025	0.418 ± 0.031	1.801	0.171	0.862	0.086	0.127
SLF_L	33:32:35	0.445 ± 0.037	0.431 ± 0.043	0.419 ± 0.048	3.121	**0.049**	**0.014**	0.273	0.179
SLF_R	33:31:35	0.460 ± 0.043	0.475 ± 0.031	0.479 ± 0.054	1.619	0.203	0.089	0.727	0.188
UF_L	33:32:35	0.400 ± 0.038	0.403 ± 0.043	0.398 ± 0.038	0.117	0.890	0.801	0.630	0.822
UF_R	33:32:35	0.395 ± 0.040	0.401 ± 0.035	0.404 ± 0.032	0.549	0.580	0.314	0.801	0.460
AF_L	31:31:33	0.488 ± 0.033	0.487 ± 0.028	0.481 ± 0.058	0.272	0.762	0.500	0.561	0.927
AF_R	29:29:34	0.472 ± 0.031	0.460 ± 0.034	0.469 ± 0.045	0.731	0.485	0.754	0.375	0.249

Notes: Data are presented as the means ± standard deviations. Abbreviations: HZ, herpes zoster; PHN, postherpetic neuralgia; HC, healthy control; F-values and *p*-values in the ANOVA column indicate ANOVA test results; FDR, false discovery rate; ATR, anterior thalamic radiation; CST, corticospinal tract; CGC, cingulum cingulate; CGH, cingulum hippocampus; CCF, corpus callosum forceps; IFOF, inferior fronto-occipital fasciculus; ILF, inferior longitudinal fasciculus; SLF, superior longitudinal fasciculus; UF, uncinated fasciculus. AF, arcuate fasciculus. L, left; R, right; P, posterior; A, anterior; n1:n2:n3 means the successfully traced number of HZ, PHN, and HC subjects, respectively, in each fiber. Bold numbers mean *p*-value < 0.05.

**Table 3:** Comparison of the mean MD among the three groups.

	n1:n2:n3	HZ	PHN	HC	ANOVA		Post Hoc Groupwise (FDR)		
		(N = 33)	(N = 32)	(N = 35)	F-Value	*p*-Value	HZ vs. HC	PHN vs. HC	HZ vs. PHN
ATR_L	33:31:35	0.765 ± 0.061	0.749 ± 0.048	0.740 ± 0.046	2.026	0.137	**0.048**	0.456	0.233
ATR_R	32:32:35	0.757 ± 0.058	0.759 ± 0.057	0.742 ± 0.053	0.960	0.387	0.269	0.212	0.887
CST_L	33:32:35	0.747 ± 0.030	0.751 ± 0.030	0.733 ± 0.023	4.133	**0.019**	**0.042**	**0.007**	0.488
CST_R	33:32:35	0.748 ± 0.037	0.757 ± 0.032	0.742 ± 0.034	1.646	0.198	0.514	0.075	0.261
CGC_L	32:32:32	0.768 ± 0.048	0.765 ± 0.044	0.736 ± 0.041	5.143	**0.008**	**0.005**	**0.009**	0.847
CGC_R	31:32:31	0.738 ± 0.049	0.749 ± 0.036	0.720 ± 0.040	3.827	**0.025**	0.085	**0.008**	0.330
CGH_L	13:10:11	0.800 ± 0.058	0.766 ± 0.067	0.800 ± 0.061	1.097	0.347	0.969	0.219	0.189
CGH_R	19:23:18	0.784 ± 0.064	0.772 ± 0.074	0.767 ± 0.059	0.308	0.736	0.451	0.813	0.575
CCF_P	33:32:35	0.872 ± 0.104	0.898 ± 0.079	0.879 ± 0.089	0.728	0.486	0.735	0.396	0.244
CCF_A	33:32:35	0.802 ± 0.058	0.810 ± 0.063	0.786 ± 0.039	1.743	0.180	0.212	0.073	0.576
IFOF_L	33:32:35	0.824 ± 0.058	0.826 ± 0.039	0.808 ± 0.036	1.455	0.238	0.166	0.129	0.883
IFOF_R	33:31:33	0.810 ± 0.047	0.823 ± 0.049	0.802 ± 0.042	1.789	0.173	0.453	0.063	0.257
ILF_L	33:31:34	0.809 ± 0.066	0.814 ± 0.048	0.794 ± 0.037	1.407	0.250	0.220	0.115	0.710
ILF_R	32:32:35	0.789 ± 0.050	0.804 ± 0.036	0.776 ± 0.041	3.643	**0.030**	0.218	**0.008**	0.157
SLF_L	33:32:35	0.752 ± 0.058	0.747 ± 0.043	0.716 ± 0.035	5.954	**0.004**	**0.002**	**0.008**	0.642
SLF_R	33:31:35	0.731 ± 0.066	0.729 ± 0.030	0.717 ± 0.037	0.851	0.430	0.225	0.327	0.831
UF_L	33:32:35	0.786 ± 0.063	0.798 ± 0.049	0.775 ± 0.036	1.633	0.195	0.374	0.071	0.358
UF_R	33:32:35	0.796 ± 0.060	0.792 ± 0.031	0.774 ± 0.038	2.337	0.102	**0.048**	0.098	0.754
AF_L	31:31:33	0.730 ± 0.041	0.741 ± 0.034	0.721 ± 0.033	2.510	0.087	0.294	**0.027**	0.246
AF_R	29:29:34	0.722 ± 0.035	0.728 ± 0.031	0.714 ± 0.035	1.406	0.251	0.331	0.101	0.513

Notes: Data are presented as the means ± standard deviations. Abbreviations: HZ, herpes zoster; PHN, postherpetic neuralgia; HC, healthy control; F-values and *p*-values in the ANOVA column indicate ANOVA test results; FDR, false discovery rate; ATR, anterior thalamic radiation; CST, corticospinal tract; CGC, cingulum cingulate; CGH, cingulum hippocampus; CCF, corpus callosum forceps; IFOF, inferior fronto-occipital fasciculus; ILF, inferior longitudinal fasciculus; SLF, superior longitudinal fasciculus; UF, uncinated fasciculus. AF, arcuate fasciculus. L, left; R, right; P, posterior; A, anterior; n1:n2:n3 means the successfully traced number of HZ, PHN, and HC subjects, respectively, in each fiber. Bold numbers mean *p*-value < 0.05.

**Table 4:** Comparison of mean RD among the three groups.

	n1:n2:n3	HZ	PHN	HC	ANOVA		Post Hoc Groupwise (FDR)		
		(N = 33)	(N = 32)	(N = 35)	F-Value	*p*-Value	HZ vs. HC	PHN vs. HC	HZ vs. PHN
ATR_L	33:31:35	0.574 ± 0.065	0.552 ± 0.045	0.550 ± 0.054	1.997	0.144	0.076	0.917	0.105
ATR_R	32:32:35	0.560 ± 0.060	0.556 ± 0.058	0.549 ± 0.057	0.348	0.707	0.411	0.618	0.752
CST_L	33:32:35	0.442 ± 0.036	0.443 ± 0.032	0.432 ± 0.031	1.100	0.337	0.236	0.181	0.870
CST_R	33:32:35	0.445 ± 0.039	0.458 ± 0.036	0.448 ± 0.040	0.941	0.394	0.769	0.304	0.195
CGC_L	32:32:32	0.554 ± 0.057	0.552 ± 0.058	0.512 ± 0.057	5.640	**0.005**	**0.004**	**0.005**	0.093
CGC_R	31:32:31	0.549 ± 0.060	0.565 ± 0.045	0.522 ± 0.043	6.107	**0.003**	**0.035**	**0.001**	0.192
CGH_L	13:10:11	0.634 ± 0.065	0.607 ± 0.065	0.637 ± 0.061	0.727	0.491	0.929	0.288	0.308
CGH_R	19:23:18	0.617 ± 0.065	0.608 ± 0.068	0.601 ± 0.064	0.288	0.751	0.453	0.736	0.649
CCF_P	33:32:35	0.522 ± 0.110	0.547 ± 0.084	0.533 ± 0.101	0.483	0.618	0.653	0.584	0.329
CCF_A	33:32:35	0.537 ± 0.067	0.547 ± 0.073	0.519 ± 0.047	1.732	0.182	0.228	0.072	0.545
IFOF_L	33:32:35	0.604 ± 0.065	0.609 ± 0.044	0.584 ± 0.039	2.404	0.096	0.091	**0.045**	0.739
IFOF_R	33:31:33	0.592 ± 0.052	0.604 ± 0.058	0.579 ± 0.045	1.849	0.163	0.327	0.058	0.343
ILF_L	33:31:34	0.619 ± 0.063	0.621 ± 0.052	0.602 ± 0.037	1.352	0.264	0.182	0.142	0.876
ILF_R	32:32:35	0.602 ± 0.054	0.620 ± 0.034	0.590 ± 0.042	3.913	**0.023**	0.244	**0.006**	0.116
SLF_L	33:32:35	0.563 ± 0.059	0.564 ± 0.048	0.546 ± 0.041	1.368	0.259	0.161	0.154	0.973
SLF_R	33:31:35	0.538 ± 0.068	0.528 ± 0.032	0.519 ± 0.053	1.022	0.364	0.156	0.482	0.491
UF_L	33:32:35	0.606 ± 0.064	0.613 ± 0.058	0.598 ± 0.035	0.609	0.546	0.566	0.273	0.602
UF_R	33:32:35	0.617 ± 0.064	0.610 ± 0.038	0.594 ± 0.041	2.069	0.132	0.050	0.177	0.552
AF_L	31:31:33	0.522 ± 0.045	0.530 ± 0.035	0.521 ± 0.054	0.356	0.702	0.958	0.448	0.487
AF_R	29:29:34	0.521 ± 0.037	0.528 ± 0.036	0.516 ± 0.047	0.737	0.482	0.665	0.231	0.459

Notes: Data are presented as the means ± standard deviations. Abbreviations: HZ, herpes zoster; PHN, postherpetic neuralgia; HC, healthy control; F-values and *p*-values in the ANOVA column indicate ANOVA test results; FDR, false discovery rate; ATR, anterior thalamic radiation; CST, corticospinal tract; CGC, cingulum cingulate; CGH, cingulum hippocampus; CCF, corpus callosum forceps; IFOF, inferior fronto-occipital fasciculus; ILF, inferior longitudinal fasciculus; SLF, superior longitudinal fasciculus; UF, uncinated fasciculus. AF, arcuate fasciculus. L, left; R, right; P, posterior; A, anterior; n1:n2:n3 means the successfully traced number of HZ, PHN, and HC subjects, respectively, in each fiber. Bold numbers mean *p*-value < 0.05.

**Table 5:** Comparison of the mean AD among the three groups.

	n1:n2:n3	HZ	PHN	HC	ANOVA		Post Hoc Groupwise (FDR)		
		(N = 33)	(N = 32)	(N = 35)	F-Value	*p*-Value	HZ vs. HC	PHN vs. HC	HZ vs. PHN
ATR_L	33:31:35	1.146 ± 0.066	1.145 ± 0.073	1.119 ± 0.049	2.020	0.138	0.078	0.098	0.937
ATR_R	32:32:35	1.151 ± 0.065	1.166 ± 0.066	1.129 ± 0.060	2.969	0.056	0.155	**0.018**	0.339
CST_L	33:32:35	1.356 ± 0.046	1.368 ± 0.056	1.333 ± 0.042	4.335	**0.016**	0.059	**0.005**	0.333
CST_R	33:32:35	1.352 ± 0.056	1.356 ± 0.045	1.331 ± 0.042	2.878	0.061	0.063	**0.029**	0.735
CGC_L	32:32:32	1.195 ± 0.067	1.192 ± 0.065	1.184 ± 0.063	0.267	0.767	0.483	0.604	0.854
CGC_R	31:32:31	1.118 ± 0.052	1.116 ± 0.066	1.116 ± 0.060	0.009	0.991	0.919	0.978	0.897
CGH_L	13:10:11	1.133 ± 0.054	1.084 ± 0.088	1.125 ± 0.078	1.383	0.266	0.800	0.208	0.123
CGH_R	19:23:18	1.117 ± 0.078	1.101 ± 0.093	1.100 ± 0.061	0.283	0.755	0.516	0.970	0.514
CCF_P	33:32:35	1.570 ± 0.113	1.601 ± 0.090	1.571 ± 0.090	1.081	0.343	0.971	0.207	0.201
CCF_A	33:32:35	1.332 ± 0.053	1.336 ± 0.055	1.320 ± 0.055	0.776	0.463	0.359	0.242	0.796
IFOF_L	33:32:35	1.263 ± 0.065	1.260 ± 0.047	1.258 ± 0.053	0.064	0.938	0.727	0.917	0.811
IFOF_R	33:31:33	1.247 ± 0.056	1.262 ± 0.043	1.247 ± 0.055	0.856	0.428	0.979	0.252	0.263
ILF_L	33:31:34	1.190 ± 0.081	1.200 ± 0.057	1.177 ± 0.056	0.979	0.379	0.441	0.166	0.531
ILF_R	32:32:35	1.161 ± 0.058	1.172 ± 0.059	1.147 ± 0.054	1.596	0.208	0.324	0.078	0.443
SLF_L	33:32:35	1.130 ± 0.076	1.113 ± 0.064	1.056 ± 0.056	11.856	**<0.001**	**<0.001**	**0.001**	0.301
SLF_R	33:31:35	1.118 ± 0.081	1.129 ± 0.050	1.113 ± 0.044	0.584	0.560	0.736	0.290	0.473
UF_L	33:32:35	1.147 ± 0.076	1.168 ± 0.052	1.129 ± 0.063	3.018	0.053	0.256	**0.016**	0.196
UF_R	33:32:35	1.153 ± 0.067	1.157 ± 0.053	1.134 ± 0.050	1.550	0.217	0.176	0.106	0.784
AF_L	31:31:33	1.147 ± 0.048	1.163 ± 0.059	1.120 ± 0.048	5.681	**0.005**	**0.038**	**0.001**	0.233
AF_R	29:29:34	1.125 ± 0.054	1.127 ± 0.044	1.109 ± 0.034	1.560	0.216	0.157	0.118	0.884

Notes: Data are presented as the means ± standard deviations. Abbreviations: HZ, herpes zoster; PHN, postherpetic neuralgia; HC, healthy control; F-values and *p*-values in the ANOVA column indicate ANOVA test results; FDR, false discovery rate; ATR, anterior thalamic radiation; CST, corticospinal tract; CGC, cingulum cingulate; CGH, cingulum hippocampus; CCF, corpus callosum forceps; IFOF, inferior fronto-occipital fasciculus; ILF, inferior longitudinal fasciculus; SLF, superior longitudinal fasciculus; UF, uncinated fasciculus. AF, arcuate fasciculus. L, left; R, right; P, posterior; A, anterior; n1:n2:n3 means the successfully traced number of HZ, PHN, and HC subjects, respectively, in each fiber. Bold numbers mean *p*-value < 0.05.

**Table 6:** Summary of changes in the location of points with significant differences revealed in the pointwise comparison of tract profiles across 20 fiber tracts among the groups.

	Location of Changes in FA			Location of Changes in MD			Location of Changes in AD			Location of Changes in RD		
	HZ vs. HC	PHN vs. HC	HZ vs. PHN	HZ vs. HC	PHN vs. HC	HZ vs. PHN	HZ vs. HC	PHN vs. HC	HZ vs. PHN	HZ vs. HC	PHN vs. HC	HZ vs. PHN
ATR_L	-	-	P	-	-	-	-	A	-	-	-	-
ATR_R	-	-	P	-	-	-	-	A/M	-	-	-	-
CST_L	-	M	M	S	S/M/I	M/I	S	S/I	I	S	S/M	M/I
CST_R	I	-	-	S	S	-	S	S	-	I	-	-
CGC_L	A/M	A/M	-	A/M/P	A/M/P	-	-	-	-	A/M/P	A/M/P	-
CGC_R	A/M	A/M/P	P	M	A/M/P	P	-	-	-	A/M	A/M/P	P
CGH_L	-	-	-	-	-	-	-	-	M	-	-	-
CGH_R	-	-	-	-	-	-	-	-	-	-	-	-
CCF_P	-	-	-	L	-	L	L	L	L	L	-	L
CCF_A	-	-	-	L	L	-	-	-	-	-	L	-
IFOF_L	P	P	-	-	M	-	-	M	-	M/P	M/P	-
IFOF_R	-	A/P	-	A	A/P	P	-	-	-	A	A/P	P
ILF_L	-	-	-	-	A	A	-	A	-	-	A	-
ILF_R	-	M	-	-	M	-	M	-	-	-	M/P	P
SLF_L	P	-	A	A/M/P	A/M/P	-	W	A/M/P	M	-	A	-
SLF_R	-	-	P	-	P	-	-	-	-	-	-	-
UF_L	-	-	-	-	T/F	-	F	M/F	M	-	T	-
UF_R	-	-	-	-	-	-	-	-	-	-	-	-
AF_L	-	-	-	M	M/F	F	M	M/F	-	-	-	-
AF_R	-	-	-	-	T	-	T	T	-	-	-	-

Abbreviations: HZ, herpes zoster; PHN, postherpetic neuralgia; HC, healthy control; FA, fractional anisotropy; MD, mean diffusivity; AD, axial diffusivity; RD, radial diffusivity; ATR, anterior thalamic radiation; CST, corticospinal tract; CGC, cingulum cingulate; CGH, cingulum hippocampus; CCF, corpus callosum forceps; IFOF, inferior fronto-occipital fasciculus; ILF, inferior longitudinal fasciculus; SLF, superior longitudinal fasciculus; UF, uncinated fasciculus. AF, arcuate fasciculus. R, right; L, left; -, no difference between the groups; A, difference in the anterior part of the fiber; P, difference in the posterior part of the fiber; S, difference in the superior part of the fiber; I, difference in the inferior part of the fiber; M, difference in the middle part of the fiber; T, difference in the temporal lobe part of the fiber; F, difference in the frontal lobe part of the fiber; W, difference in the entire fiber.

**Table 7:** Number of points with significant differences determined by the pointwise comparison of tract profiles across 20 fiber tracts after FDR correction.

	FA			MD			AD			RD		
	HZ vs. HC	PHN vs. HC	HZ vs. PHN	HZ vs. HC	PHN vs. HC	HZ vs. PHN	HZ vs. HC	PHN vs. HC	HZ vs. PHN	HZ vs. HC	PHN vs. HC	HZ vs. PHN
ATR_L	0	0	7	0	0	0	0	5	0	0	0	0
ATR_R	0	0	8	0	0	0	0	11	0	0	0	0
CST_L	0	4	5	23	23	10	4	25	7	6	10	10
CST_R	6	0	0	6	6	0	4	4	0	3	0	0
CGC_L	21	21	0	22	30	0	0	0	0	30	32	0
CGC_R	17	38	5	3	19	3	0	0	0	17	41	4
CGH_L	0	0	0	0	0	0	0	0	10	0	0	0
CGH_R	0	0	0	0	0	0	0	0	0	0	0	0
CCF_P	0	0	0	9	0	3	3	4	3	5	0	3
CCF_A	0	0	0	4	8	0	0	0	0	0	5	0
IFOF_L	6	18	0	0	6	0	0	5	0	17	20	0
IFOF_R	0	8	0	3	13	6	0	0	0	3	13	3
ILF_L	0	0	0	0	18	3	0	4	0	0	10	0
ILF_R	0	4	0	0	4	0	7	0	0	0	7	3
SLF_L	15	0	12	54	58	0	100	78	15	0	10	0
SLF_R	0	0	10	0	11	0	0	0	0	0	0	0
UF_L	0	0	0	0	16	0	14	78	7	0	9	0
UF_R	0	0	0	0	0	0	0	0	0	0	0	0
AF_L	0	0	0	4	17	4	8	36	0	0	0	0
AF_R	0	0	0	0	15	0	4	12	0	0	0	0

Abbreviations: HZ, herpes zoster; PHN, postherpetic neuralgia; HC, healthy control; FA, fractional anisotropy; MD, mean diffusivity; AD, axial diffusivity; RD, radial diffusivity; ATR, anterior thalamic radiation; CST, corticospinal tract; CGC, cingulum cingulate; CGH, cingulum hippocampus; CCF, corpus callosum forceps; IFOF, inferior fronto-occipital fasciculus; ILF, inferior longitudinal fasciculus; SLF, superior longitudinal fasciculus; UF, uncinated fasciculus. AF, arcuate fasciculus. R, right; L, left.

## Data Availability

The data that support the findings of this study are available from the corresponding author upon reasonable request.
